# miR-602 Activates NRF2 Antioxidant Pathways to Protect HBMECs from OGD/R-Induced Oxidative Stress via Targeting KEAP1 and HRD1

**DOI:** 10.1155/2022/6967573

**Published:** 2022-09-24

**Authors:** Chunli Ma, Lei Yang, Qiang Gao, Lihua Wang

**Affiliations:** ^1^Department of Neurology, The Second Affiliated Hospital of Mudanjiang Medical College, Mudanjiang City, 157000 Heilongjiang Province, China; ^2^Clinical Skills Training Center, First Clinical Medical College, Mudanjiang Medical College, Mudanjiang, 157011 Heilongjiang, China; ^3^Department of Geriatrics, The Second Affiliated Hospital of Harbin Medical University, Harbin City, 150001 Heilongjiang Province, China; ^4^Department of Neurology, The Second Affiliated Hospital of Harbin Medical University, Harbin City, 150001 Heilongjiang Province, China

## Abstract

Blood brain barrier (BBB) dysfunction is a critical complication of diabetes mellitus type 2 (T2DM), and the oxidative stress-induced apoptosis of human brain microvascular endothelial cells (HBMECs) is a main cause of BBB dysfunction. In this study, oxygen and glucose deprivation/reoxygenation (OGD/R) models were established with HBMECs to analyze the effects of miR-602 on the apoptosis of HMBECs. Western Blot, qRT-PCR, CCK-8, flow cytometry assay, ROS detection assay, and dual-luciferase reporter gene assay were used to measure the expression levels of corresponding factors and changes in intracellular environment. The results showed that miR-602 was overexpressed in HBMECs after OGD/R treatment, and miR-602 could reduce ROS level of OGD/R-induced HBMECs and promote cells survival via increasing the expression level of NRF2 and the transcription activity of NRF2/ARE. Besides, it was found that KEAP1 and HRD1 were downstream factors of miR-602, and the increase of both KEAP1 and HRD1 could reverse the effects of miR-602 on the OGD/R-induced HMBECs. Therefore, miR-602 may be a potential target for research and treatment of the oxidative stress injury induced by apoptosis in HMBECs.

## 1. Introduction

Diabetes mellitus type 2 (T2DM) is an intractable disease induced by insulin deficiency or resistance, which seriously threatens the health of the human. The T2DM patients exhibit the disorder in blood glucose metabolism and are usually accompanied with multiple complications. Moreover, the study has showed that T2DM is related with stroke induced by the disruption of the blood brain barrier (BBB) [1, 2]. BBB is the barrier between plasma and brain that protects mammalian brain from viruses, microorganisms, chemical molecules, and other harmful proteins in blood [2]. BBB dysfunction may be associated with a variety of neurological diseases, such as Alzheimer's disease (AD), Huntington's disease (HD), and Parkinson's disease (PD) [3]. Many patients diagnosed with AD suffer from BBB dysfunction [4]. Brain microvascular endothelial cells (BMECs) are important cells in the structure of capillaries and are the major substance in the formation of BBB [5]. BMECs can be induced by vascular endothelial growth factor to form capillary wall and BBB by connecting with neuroglial cells [6]. However, abnormal apoptosis of human brain microvascular endothelial cells (HBMECs) has been found to induce BBB dysfunction [7–9]. Ischemia/reperfusion is the direct cause of oxidative stress in brain cells, which can induce oxidative damage of the related proteins and DNA, and eventually lead to the apoptosis of neuronal cell and brain injury [10, 11]. Oxidative stress has been proved to be one of the important factors in inducing apoptosis of HBMECs [12]. Therefore, understanding the molecular mechanism of oxidative stress induced by cerebral ischemia/reperfusion in HBMECs can provide novel therapeutic approaches for the treatment of diseases related to BBB.

MicroRNAs (miRNAs) are a class of endogenous small noncoding RNA that directly target the 3′-untranslated regions (3′-UTR) of the messenger RNAs (mRNAs) and finally affect the expression levels of the related proteins [13]. miRNAs are associated with some central nervous system diseases, such as AD and stroke [14]. Considering the mechanism of miRNAs, the novel therapeutic strategies have been widely used in the treatment and research of human diseases [15]. A study has found that miR-602 is downregulated in the serum of the patients with ischemic stroke, and miR-602 can be as a potential biomarker for the diagnosis of ischemic stroke [16]. Besides, we have also found that miR-602 is overexpressed in HBMECs under oxidative stress situation induced by oxygen and glucose deprivation/reoxygenation (OGD/R) treatment in preliminary work of this study.

In this study, the OGD/R cell model was used to explore the role of miR-602 in inhibiting oxidative stress injury of HBMECs and is aimed at providing reference for the research and treatment of the neurological complication induced by T2DM.

## 2. Materials and Methods

### 2.1. Participants

Fifteen patients were included in the study with the diagnosis of the acute ischemic stroke (IS) based on clinical features according to the World Health Organization definition and supported by brain imaging (CT or MRI). The control group included 15 age and sex matched healthy individuals without a history of IS. Blood samples were collected at admission for RT-qPCR. Patients or family members have signed informed consent. This study was approved by the hospital ethics committee.

### 2.2. Cell Lines and Cell Culture

HBMECs lines (BeNa Culture Collection, Beijing, China) were cultured in Endothelial Cell Medium (HyClone Logan, State of Utah, USA) containing 5% fetal bovine serum (FBS). Cells were incubated with 5% CO_2_ in humidified incubator at 37°C. Trypsinase solution (0.25%) (HyClone Logan, State of Utah, USA) was used to obtain adherent cells.

### 2.3. Cell Transfection

The miR-602 mimics, miR-602 inhibitors, siRNA-NC, NRF2-siRNA, pcDNA-KEAP1, and HRD1-siRNA were designed and provided by the Generay Biotech (Shanghai) Co., Ltd. (Shanghai, China). HBMECs (2 × 10^6^ cells/well) were seeded in 6-well plates and incubated in complete medium for 24 hours. After that, the miR-602 mimics, miR-602 inhibitors, NRF2-siRNA, siRNA-NC, pcDNA-KEAP1, or HRD1-siRNA were transfected into HBMECs using Lipofectamine 2000 (Invitrogen, California, USA), and then all cells were incubated in an incubator with 5% CO_2_ at 37°C for 48 hours. The negative control (NC) of miR-602 had no significant homology with any known human sequences.

### 2.4. OGD/R Treatment [17]

OGD intervention was conducted as previously described [18]. HBMECs were used to establish the OGD/R cell models. HBMECs were seeded in 6-well plates and incubated until 70% confluence. Subsequently, the cells were cultured with glucose-free medium at the hypoxic conditions containing 85% N_2_, 10% CO_2_, and 5% O_2_ for 6 hours. After that, the cells were further cultured with complete medium in a humidified incubator at 37°C, 5% O_2_ and 95% air for 12 hours or 24 hours.

### 2.5. RNA Extraction and RT-qPCR Analysis

Total RNA was extracted with TRIzol reagent, and then cDNA was synthesized using the Revert Aid First Strand cDNA Synthesis Kit (Thermo Fisher, Massachusetts, USA). The primers of miR-602 and Brf2 were synthesized and purified by RiboBio (Guangzhou, China). qRT-PCR were performed with 7300 Real-Time PCR System (Applied Biosystems, Waltharm, MAm USA). The following conditions were used: denaturation at 95°C for 3 min, followed by amplification for 40 cycles at 95°C for 12 s and at 62°C for 40 s, and 70°C for 30 s. The relative expression levels of miRNAs was calculated with the 2^−(ΔΔCt)^ method. U6 was used as the endogenous controls. The primer sequences are listed in Table 1 [19, 20].

### 2.6. Western Blot

The total protein of HBMECs was extracted with RIPA buffer and 1% PMSF (Beyotime, Shanghai, China) for Western Blot. The concentration of proteins was measured by using a Pierce BCA protein assay kit (Beyotime, Shanghai, China). The proteins in the extracts were separated by 10% SDS-PAGE gels, and then were transferred from SDS-PAGE to PVDF membranes. After sealing with 5% skim milk for 1 hour, the membranes were incubated with the primary antibodies overnight at 4°C. After washing with TBST for three times, the membranes were incubated with the secondary antibodies for 1.5 hours. Finally, a chemiluminescence detection system was used to observed protein samples. The antibodies were used as follow: anti-NRF2 (68 kDa, 1 : 1000, ab62352, Abcam); anti-KEAP1(70 kDa, 1 : 1000, ab139729, Abcam); anti-HRD1 (67 kDa, 1 : 2000, ab170901, Abcam); anti-MCM2 (102 kDa, 1 : 2000, ab108935, Abcam); and anti-*β*-actin (42 kDa, 1 : 1000, sc-47,778, Santa Cruz).

### 2.7. Dual-Luciferase Reporter Gene Assay

The pmirGLO luciferase reporter vectors containing the 3′-UTR sequence of wild type or mutant type of KEAP1 were named as KEAP1-wt and KEAP1-mut, respectively. The pmirGLO luciferase reporter vectors containing the 3′-UTR of wild type or mutant type of HRD1 were named as HRD1-wt and HRD1-mut, respectively. The mutant sequences were designed following the binding sites in Table 2. The luciferase vectors of KEAP1 or HRD1 were cotransfected into HBMECs with miR-602 mimics or miR-NC, and the cells were incubated for 48 hours. Finally, the luciferase activity of HBMECs was observed by dual-luciferase reporter assay system.

### 2.8. Flow Cytometry Assay

The HBMECs were harvested after treatment with trypsinase (0.25%, EDTA-free), and after washing with ice phosphate-buffered saline (PBS) for three times, 2 × 10^3^ cells were diluted in ice Annexin V-FITC binding buffer. Subsequently, HBMECs were incubated with Annexin V-FITC under the darkness for 10 min, and after incubating with propidium iodide (PI) at room temperature, the apoptosis levels of the cells were instantly observed by flow cytometry equipment (BD Biosciences, State of New Jersey, USA).

### 2.9. CCK-8

HBMEC cells were seeded into 96-well plates with 5 × 10^4^ cells/well. After transfection, the cells were incubated for 48 hours, and then were induced by OGD/R treatment. Subsequently, 10 *μ*L of CCK-8 solution (Amyjet, Wuhan, China) was added to each well, and the cells were incubated for 2 hours at 37°C for 2 hours. The absorbance value was measured by a microplate reader (Flash, Shanghai, China) at 450 nm.

### 2.10. ROS Detection Assay

HBMECs were seeded in 96-well plates at 2 × 10^4^ cells per well. The ROS level of HBMEC was measured by DCFH-DA assay. HBMECs were cultured in serum-free medium and loaded with DCFH-DA at a final concentration of 10 *μ*m. After incubation under the darkness at 37°C for 20 min, the cells were washed by serum-free medium. After that, the absorbance was measured by multifunctional microplate detector. The excitation wavelength was 488 nm, and the emission wavelength was 525 nm.

### 2.11. Detection of NRF2 Transcription Activity

The pARE luciferase reporter vectors containing the binding sites of ARE were cotransfected with KEAP1 or HRD1 expression vectors into HBMECs, and then the cells were incubated for 48 hours. In addition, the pRL Renilla luciferase control reporters were used as control. After that, the cells were incubated under the darkness for 48 hours. After OGD/R treatment, the transcription activity of the cells was observed by luciferase reporter assay system (Promega, Wisconsin, USA).

### 2.12. Statistical Analysis

The data were shown as mean ± SD. The data were analyzed and displayed by SPSS 19.0 and GraphPad Prism 8, respectively. The difference of all groups was calculated through the one-way ANOVA with Tukey's post hoc test. All of the experiments were performed independently at least three times. A value of *P* < 0.05 was considered to be statistically significant.

## 3. Results

### 3.1. miR-602 Was Overexpressed in OGD/R Cell Models

**Figure 1:**
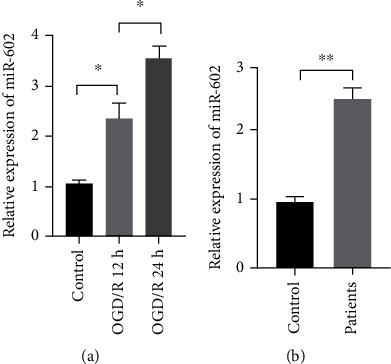
The miR-602 expression was obviously raised in HBMECs after OGD/R exposure. HBMECs were subjected to OGD treatment for 6 hours, and then followed by reoxygenation in normal medium for 12 and 24 hours. The relative expression of miR-602 was measured by qRT-PCR in HBMECs after OGD/R exposure (a) and serum in BBB (b). ^∗∗^*P* < 0.01.

To analyze the relationship of miR-602 and oxidative stress-induced damage of HBMECs, the OGD/R model established by HBMECs was used for subsequent experiments. qRT-PCR was used to measure the expression level of miR-602 after OGD/R treatment for 12 and 24 hours. The results showed that miR-602 was significantly upregulated in HBMECs induced by OGD/R treatment. Besides, it was also found that the expression level of miR-602 was higher in the cells induced by OGD/R treatment for 24 hours than that in the OGD/R-induced HBMECs for 12 hours (Figure 1(a), *P* < 0.01). In addition, the expression of miR-602 in serum of IS patients was obviously higher than that in healthy people (Figure 1(b), *P* < 0.01).

### 3.2. miR-602 Deficiency Enhanced the Oxidative Damage of HBMECs Induced by OGD/R Treatment

**Figure 2:**
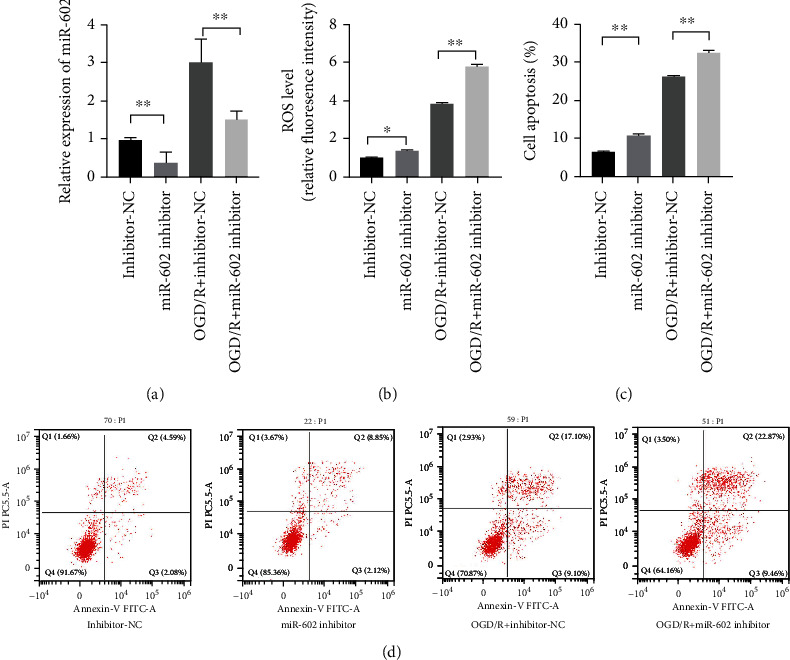
MiR-602 deficiency exacerbated the injury induced by OGD/R treatment on HBMECs. HBMECs were transfected with miR-602 inhibitor for 48 hours and then subjected to OGD/R treatment. (a) The relative expression level of miR-602. (b) The effect of miR-602 deficiency in ROS level in HBMECs was detected by ROS detection assay. (c) The effect of miR-602 deficiency on the viability of HBMECs was detected by CCK-8. (d) The effect of miR-602 deficiency on the apoptosis was observed by flow cytometry assay. ^∗^*P* < 0.05, ^∗∗^*P* < 0.01.

To explore the role of miR-602 in oxidative stress-induced damage of HBMECs, miR-602 inhibitors were transfected into HBMECs before OGD/R treatment, and the effect of miR-602 on OGD/R-induced HBMECs was verified by qRT-PCR. ROS detection assay and flow cytometry assay were used to observe the ROS level and cell apoptosis rate of HBMECs, respectively. The results showed that compared with the non-OGD/R groups, the ROS level of the cells after OGD/R treatment was increased significantly, and the ROS level in the cell transfected with miR-602 inhibitor was significantly higher than that in the cells with low miR-602 expression level (Figures 2(a) and 2(b), *P* < 0.01). The flow cytometry assay and CCK-8 demonstrated that OGD/R treatment could promote the apoptosis level and decrease the viability of HBMECs, and miR-602 deficiency could aggravate those phenomena (Figures 2(c)–2(e), *P* < 0.01). In short, these results suggested that the depletion of miR-602 enhanced the damage of HBMECs induced by OGD/R treatment.

### 3.3. miR-602 Overexpression Alleviated Oxidative Damage of HBMECs Induced by OGD/R Treatment

**Figure 3:**
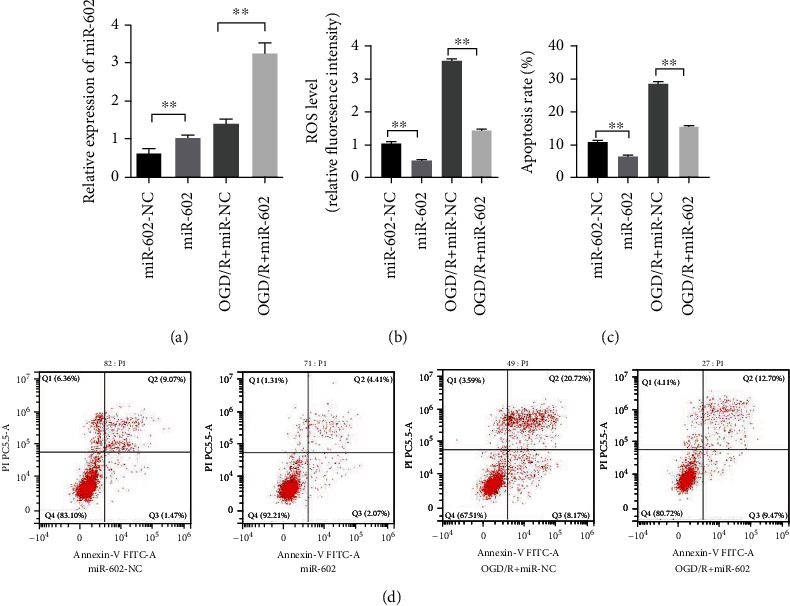
MiR-602 upregulation alleviated OGD/R-induced injury of HBMECs. HBMECs were transfected with miR-602 inhibitor for 48 hours and then subjected to OGD/R treatment. (a) The relative expression level of miR-602. (b) The effect of miR-602 upregulation on the ROS level of HBMECs was detected by ROS detection assay. (c, d) The effect of miR-602 upregulation on the apoptosis of HBMECs was observed by flow cytometry assay. ^∗∗^*P* < 0.01.

The effects of miR-602 depletion on HBMECs oxidative damage was proved above. To investigate whether miR-602 could promote HBMEC survival under OGD/R exposure, the miR-602 mimics were transfected into HBMECs before OGD/R treatment, and ROS detection assay and flow cytometry assay were used to observe the ROS level and cell apoptosis rate, respectively. The ROS detection assay showed that high expression level of miR-602 could effectively reverse the ROS level of HBMECs after OGD/R treatment for 24 hours (Figures 3(a) and 3(b), *P* < 0.01). Flow cytometry assay manifested that the apoptosis rate of HBMECs was increased significantly after OGD/R treatment for 24 hours, whereas overexpressed miR-602 could remarkably decrease the apoptosis rate of OGD/R-induced HBMECs (Figures 3(c) and 3(d), *P* < 0.01). Therefore, these results suggested that miR-602 played a positive role in inhibiting the apoptosis of HBMECs during oxidative stress-induced damage.

### 3.4. miR-602 Alleviated HBMECs Oxidative Damage via NRF2

**Figure 4:**
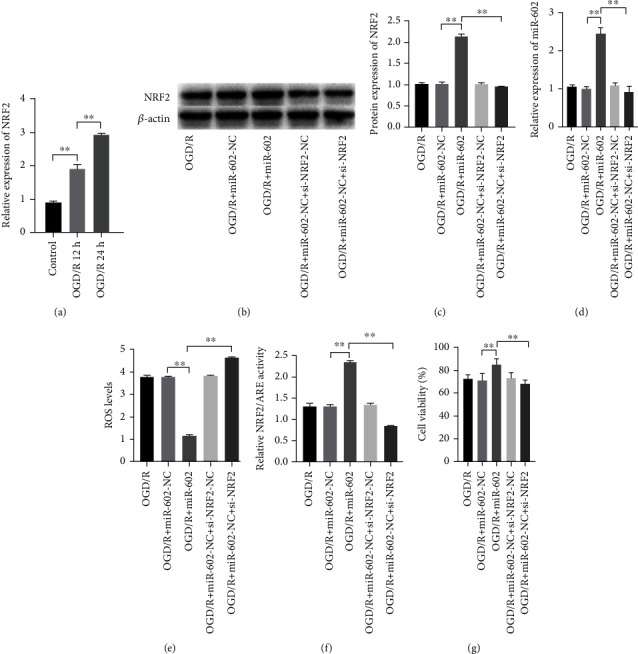
NRF2 silence could reverse the effect of miR-602 on decreasing the injury of HBMECs induced by OGD/R. HBMECs were transfected with miR-602 mimic and si-NRF2 for 48 hours and then subjected to OGD/R treatment. (a) The effect of OGD/R on the expression of NRF2. (b, c) The relative expression level of NRF2. (d) The relative expression level of miR-602. (e) The effect of NRF2 silence on the ROS level of HBMECs when miR-602 was upregulated. (f) The effect of NRF2 silence on the relative NRF2/ARE transcription activity in HBMECs when miR-602 was upregulated. (g) The effect of NRF2 silence on the viability of HBMECs when miR-602 was upregulated. ^∗∗^*P* < 0.01.

To excavate the association between miR-602 and HBMECs oxidative damage, antioxidant related factors in cells such as NRF2 were selected as the research objects. Western Blot was used to detect protein level of NRF2 and dual-luciferase reporter assay was used to detect NRF2/ARE transcription activity in OGD/R-induced HBMECs when miR-602 was overexpressed. Compared with the NC-transfected cells, the NRF2 was significantly upregulated in the cells induced with OGD/R treatment when miR-602 was overexpressed. Contrarily, NRF2 was significantly downregulated in the cells transfected with miR-602 inhibitor (Figures 4(a)–4(c), *P* < 0.01). In addition, when miR-602 was downregulated in the cells, NRF2/ARE showed weak transcription activity, whereas the situation was opposite when miR-602 was at high expression level (Figure 4(e), *P* < 0.01). These results suggested that the role of miR-602 was related to the KEAP1/NRF2 signaling pathway.

### 3.5. NRF2 Inhibition Reserved miR-602 Protective Effects on HBMECs

To verify the association between NRF2 and the protective effects of miR-602 on HBMECs after OGD/R treatment, si-NRF2 and miR-602 were cotransfected into HBMECs. ROS detection assay, luciferase reporter assay, and CCK-8 were used to observe ROS level, NRF2/ARE activity, and apoptosis rate, respectively. The results showed that the cells with high expression level of NRF2 had high ROS level, NRF2/ARE activity and apoptosis rate (Figures 4(d), 4(f), and 4(g), *P* < 0.01 for all). It was found that miR-602 upregulation could significantly decrease the ROS level, NRF2/ARE activity, and apoptosis rate, whereas those effects of miR-602 on OGD/R-induced HBMECs could be reversed by NRF2 downregulation (Figures 4(d) and 4(e), *P* < 0.01 for all). Those results suggested that the effects of miR-602 on protecting HBMECs from oxidative damage depend on high expression level of NRF2.

### 3.6. miR-602 Regulated NRF2 Expression via Targeting KEAP1

**Figure 5:**
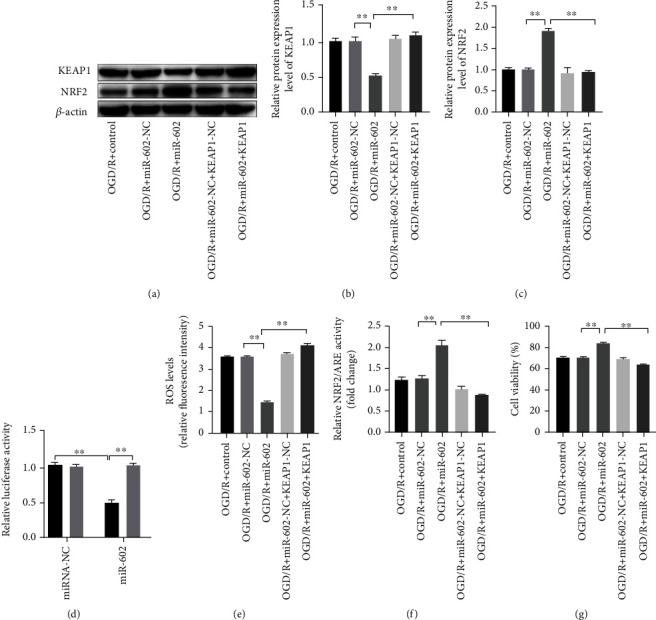
KEAP1 overexpression reversed the effect of miR-602 on decreasing the injury of HBMECs induced by OGD/R. HBMECs were transfected with miR-602 mimic and KEAP1 expressed vector for 48 h and then subjected to OGD/R treatment. (a, b, c) The relative expression levels of NRF2 and KEAP1. (d) Binding effect of KEAP1 and miR-602 was observed by dual-luciferase reporter assay. (e) The effect of KEAP1 upregulation on the ROS level of HBMECs when miR-602 was upregulated. (f) The effect of KEAP1 upregulation on the relative NRF2/ARE transcription activity in HBMECs when miR-602 was upregulated. (g) The effect of increased KEAP1 on the viability of HBMECs when miR-602 was upregulated. ^∗∗^*P* < 0.01.

To further illuminate mechanism of miR-602 in regulating NRF2 expression, miRWalk, an online microRNAs targets prediction database (http://mirwalk.umm.uni-heidelberg.de/), was used to predict the targets of miR-602. According to the predicted results, KEAP1 was selected as a candidate of miR-602 for the next experiments. The dual-luciferase reporter assay showed that miR-602 repressed the luciferase activity of KEAP1-wt, but had no effect on KEAP1-mut (Figure 5(d)). After that, Western Blot was used to measure protein expression level of KEAP1 and NRF2. The results reflected that the protein expression level of KEAP1 was decreased significantly when miR-602 was overexpressed in HBMECs (Figures 5(a)–5(c)). To further confirm the effect of KEAP1 on the pathway of miR-602 mediating NRF2 activation, KEAP1 and miR-602 mimic were cotransfected into the cells to observe changes of the ROS level, NRF2/ARE transcription activity, and apoptosis rate of OGD/R-induced HBMECs. The results showed that the ROS level, transcription activity of NRF2/ARE, and cell viability of the cells induced by miR-602 could be reversed by KEAP1, significantly (Figures 5(e)–5(g), *P* < 0.01 for all).

### 3.7. miR-602 Promoted NRF2 Expression via Regulating the Expression of HRD1

**Figure 6:**
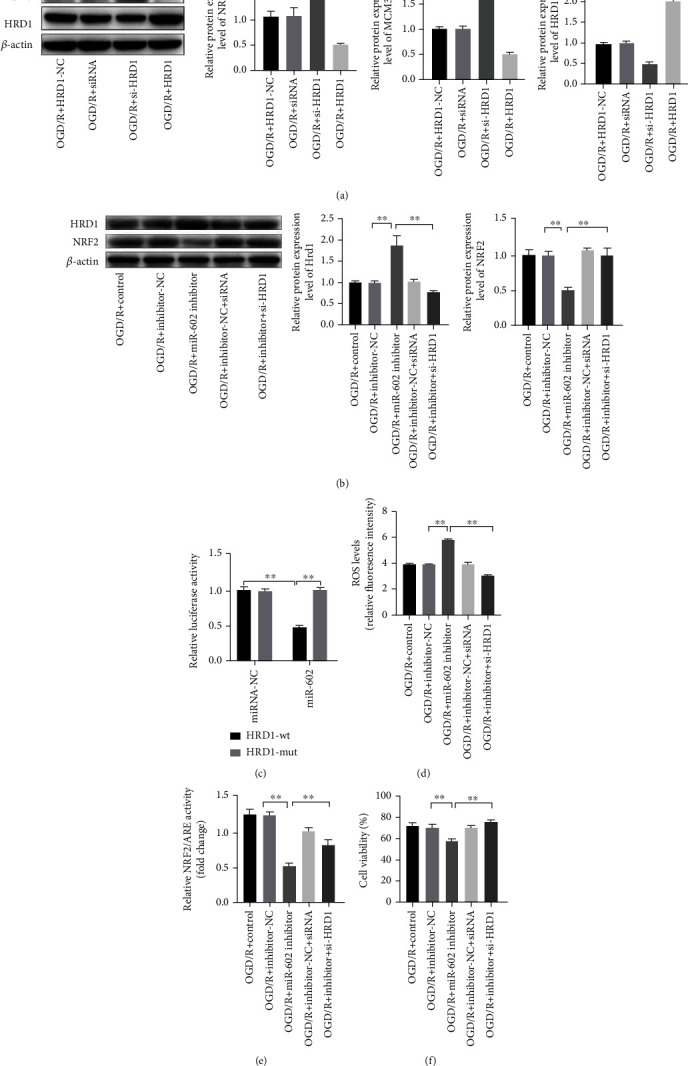
HRD1 Silence could reserve OGD/R-induced injury to HBMECs at miR-602 deficiency situation. HBMECs were transfected with mimic of miR-602 inhibitor and HRD1 for 48 hours and then subjected to OGD/R treatment. (a) NRF2 and MCM3 relative expression level were detected by Western Blot. (b) NRF2 and HRD1 relative expression level at miR-602 deficiency situation was detected by Western Blot. (c) Luciferase activity of HRD1 3′-UTR wt and HRD1 3′-UTR mut with miR-602. (d) The effect of HRD1 silence on ROS level of HBMECs with low miR-602 level was detected by ROS detection assay. (e) The effect of HRD1 silence on relative NRF2/ARE transcription activity in HBMECs with low miR-602 level was detected by luciferase reporter assay. (f) The effect of HRD1 silence on the viability of HBMECs with low miR-602 level was detected by CCK-8. ^∗∗^*P* < 0.01.

According to prediction results above, it was hypothesized that HRD1 was also found as a potential target of miR-602. To validate this hypothesis, dual-luciferase reporter gene assay was applied to verify the combination effect of miR-602 on HRD1, and the result showed that miR-602 mimics repressed the luciferase activity of HRD1-wt, but had no effect on HRD1-mut (Figure 6(c)). After that, Western Blot was used to measure expression levels of HRD1, MCM3, and NRF2. The results reflected that HRD1 was significantly downregulated, but MCM3 and NRF2 were significantly upregulated in OGD/R-induced HBMECs when miR-602 was overexpressed (Figures 6(a) and 6(b)). To further confirm the role of HRD1 in miR-602 mediating NRF2 activation, the si-HRD1 and miR-602 inhibitor were cotransfected into the cells to observe the effects of HRD1 on the ROS level, NRF2/ARE transcription activity, cell viability, and apoptosis rate of OGD/R-induced HBMECs. The results showed that compared with the miR-602 inhibitor groups, HRD1 downregulation in large degree reversed the effects of miR-602 deficiency on the oxidative stress-induced damage of HBMECs (Figures 6(d)–6(f), *P* < 0.01 for all).

## 4. Discussion

T2DM has been regarded to involve multiple complications, such as dementia, cerebrovascular disease, and depression, and increasing researchers have focused on intervening metabolism dysfunction-mediated neurological diseases to improve the prognosis of the patients with T2DM. BBB injury is a critical complication of T2DM, and serious BBB disruption can impair the normal running of central nervous system [20]. Oxidative stress-induced apoptosis of HBMECs is one of the main causes of BBB dysfunction. The OGD/R cell model established by HBMECs is often used to imitate the brain environment and explore the pathogenesis of BBB [21, 22]. In this study, the OGD/R cell model was also established to observe the effects of oxidative stress on HBMECs, and the exploration of the association between miR-602 and OGD/R-induced HBMECs was also performed. This study demonstrated that miR-602 was essential for protecting HBMECs from oxidative damage induced by OGD/R, and miR-602 could affect the transcription of NRF2 via regulating the abundance of KEAP1 to protect HBMECs from oxidative damage. Besides, we also confirmed that HRD1 was the target of miR-602, and miR-602 could promote the combination of NRF2 and KEAP1 via inhibiting the expression of MCM3.

Several studies have indicated that miR-602 is associated with a variety of cancers and involves in the formation and development of these cancers by regulating some key factors and pathways. For instance, miR-602 was thought as a tumor oncogene which could promote cancer cell growth and metastasis by regulating FOXK2 in esophageal squamous cell carcinoma [23]. However, this study demonstrated the protective effects of miR-602 on oxidative damage of HBMECs induced by OGD/R. In this study, we detected the changes of miR-602 levels in the OGD/R cell model and normal HBMECs, and found that miR-602 was significantly upregulated in the HBMECs induced by OGD/R. One study has indicated that miR-602 is at low level in the serum of the patients with ischemic stroke. According to knockdown and overexpression experiments, we explored the role of miR-602 in the OGD/R cell model. Therefore, we believe that miR-602 can protect HBMECs from the oxidative stress-induced damage via decreasing the ROS level and cell apoptosis.

NRF2 has been proved as a potentially promising target against oxidative stress damage in cerebral ischemia stoke [24]. The expression level of NRF2 in brain tissue is closely related to AD, and the decrease of NRF2 and its target genes has been found in AD transgenic animals models [25, 26]. Besides, high expression level of NRF2 in hippocampus also has a positive effect on improving the cognitive ability of APP/PS1 mice [27]. In this study, we revealed that NRF2 was a key factor of miR-602 on protecting HBMECs from oxidative stress damage induced by OGD/R treatment. Subsequently, we determined that KEAP1 was one of the credible targets of miR-602. KEAP1 is an ubiquitination facilitator protein, which can mediate ubiquitination of NRF2 and further restrict the transcription activation of NRF2/ARE via tightly binding with NRF2 [28]. According to the rescue experiments, it was found that KEAP1 could reverse the effects of miR-602 on OGD/R-induced HBMECs. KEAP1/NRF2 signaling pathway was found to take part in several neurogenic diseases, and several studies have confirmed that increased NRF2 induced by inhibited KEAP1 could significantly alleviate oxidative stress damage to some diseases [29, 30]. Besides, we also found that HRD1 was one of the potential targets of miR-602. HRD1 has been verified as an E3 ubiquitin ligase, which can suppress expression of NRF2 to promote liver cirrhosis development [31, 32]. We determined that the downregulation of HRD1 could also reserve the high ROS and apoptosis levels of HBMECs induced by miR-602 deficiency. Interestingly, we found that HRD1 could connect with MCM3. One study has indicated that NRF2 has similar structure with conserved helix-2-insert motif of MCM3, and thus MCM3 can increase the expression level of NRF2 via combining with KEAP1 in a way of competitive binding [33]. In this study, notable change of MCM3 level was also observed in the OGD/R cell models when HRD1 was upregulated.

In summary, this study suggests that miR-602 can reduce ROS and apoptosis level and activate the transcription activity of NRF2/ARE to protect HBMECs from the oxidative stress induced by OGD/R via targeting KEAP1 and HRD1. Given the effect of NRF2 on the apoptosis pathway of HBMECs and the association between BBB dysfunction and HBMECs apoptosis, we believe that treating miR-602 as a target to regulate apoptosis of HBMECs may represent a feasible approach for the treatment of BBB dysfunction related diseases. However, more credible evidence of miR-602/KEAP1/NRF2 axis and miR-602/HRD1/NRF2 axis in OGD/R-induced HBMECs needs to be demonstrated by in vivo animal model experiments.

## Figures and Tables

**Table 1:** Primer sequence of miR-602, NRF2, and U6.

Name of primer	Sequences
miR-602-F	5′-TCGGCAGGGACACGGGCGACAG-3′
miR-602-R	5′-CTCAACTGGTGTCGTGGA-3′
NRF2-F	5′-TCCAGTCAGAAACCAGTGGAT-3′
NRF2-R	5′-GAATGTCTGCGCCAAAAGCTG-3′
U6-F	5′-CTCGCTTCGGCAGCACA-3′
U6-R	5′-AACGCTTCACGAATTTGCGT-3′

**Table 2:** The binding sites of miR-602 and its related targets.

Name of primer	Sequences
miR-602	5′-GACACGGGCGACAGCUGCGGCCC-3′
HRD1-wt	3′-......TCGGTTTGTGGTCGACGTGTC..-5′
HRD1-mut	3′-......TCGGTTTGTGCAGCUGCTGTC..-5′
miR-602	5′-GACACGGGCGACAGCUGCGGCCC-3′
KEAP1-wt	3′-.......TGTACCACTGTCGACGGCCGT..-5′
KEAP1-mut	3′-.......TGTACCAGACAGCUGCGCCGT..-5′

## Data Availability

Data to support the findings of this study is available on reasonable request from the corresponding author.
